# Supplementary material for: Microglia-neuron interaction at nodes of Ranvier depends on neuronal activity through potassium release and contributes to remyelination
Source: Nat Commun. 2021 Sep 1;12:5219. doi: 10.1038/s41467-021-25486-7 (PMC8410814; doi:10.1038/s41467-021-25486-7)
Supplement: Supplementary file 1 — Supplementary Information [file 41467_2021_25486_MOESM1_ESM.pdf]

Supplementary Figures

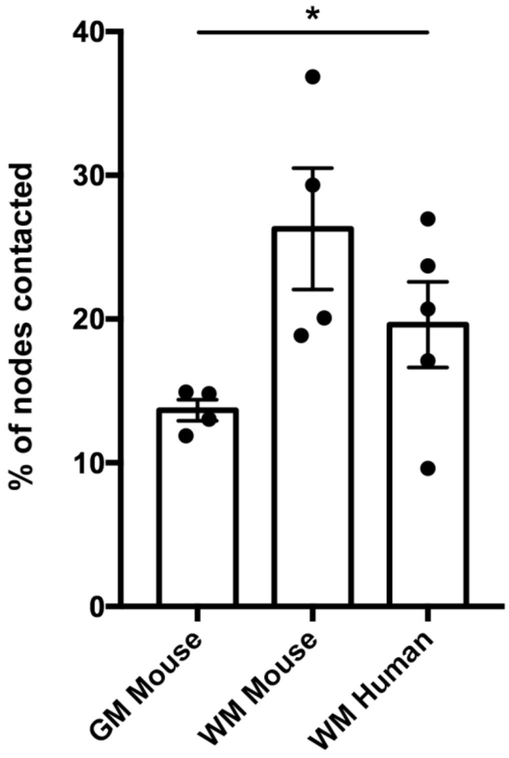

Figure S1: Percentage of nodes contacted by microglial cells in central nervous tissues. GM Mouse = mouse grey matter (cerebellum); WM Mouse = mouse white matter (spinal cord) and WM Human: human hemispheric white matter. The mean values per individual are plotted as dots. Bars and error bars represent the mean ± s.e.m. Two-sided Kruskal-Wallis test. For detailed statistics, see Supplementary Table.

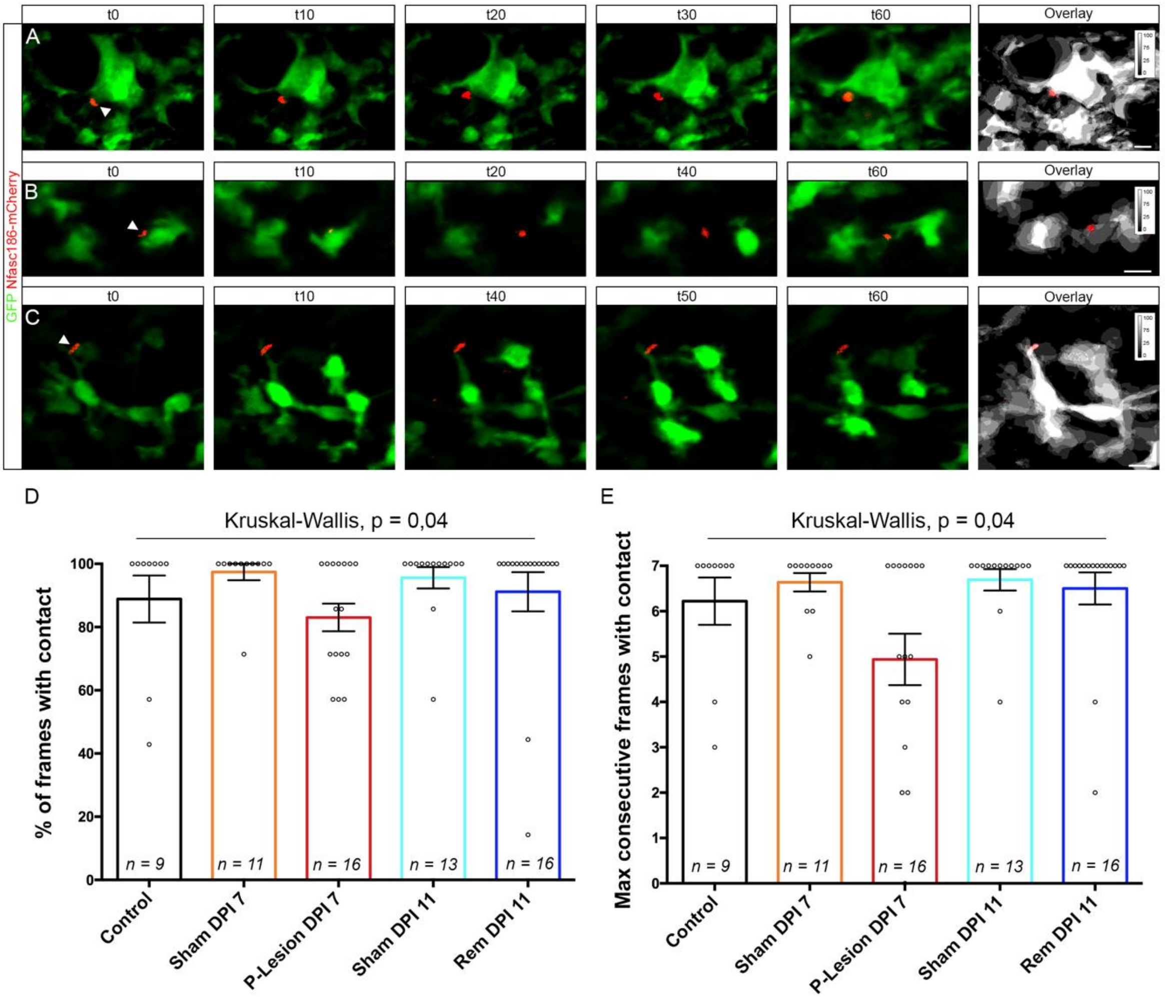

Figure S2. Microglial contacts with nodes of Ranvier are less stable during demyelination. (A-C): *In vivo* live-imaging from CX3CR1-GFP/Thy1-Nfasc186mCherry mouse dorsal spinal cord with an initial contact between microglia (green) and nodes of Ranvier (red, arrowheads); 1-hour movies with an acquisition every 10 minutes. Scale bar 10 µm; (A): Sham animal (NaCl injection) imaging at 7 DPI (corresponding to Movie 2 and condition Sham DPI7, n=11 animals); (B): LPC-injected animal imaged at 7 DPI (demyelination, corresponding to Movie 3 and P-Lesion DPI7, n=16 animals); (C): LPC-injected animal at 11 DPI (remyelination, corresponding to Movie 4 and Rem DPI11, n=16 animals). (D) Percentage of frames with microglia-node contact in 3-hour movies. (E) Longest sequence of consecutive timepoints with microglia-node contact in 3-hour movies. Each dot is a microglia-node pair. The number of microglia-node pairs imaged is indicated on each bar (n=4 to 7 animals per condition). Two-sided Kruskal-Wallis test. Bars and error bars represent the mean ± s.e.m. For detailed statistics, see Supplementary Table.

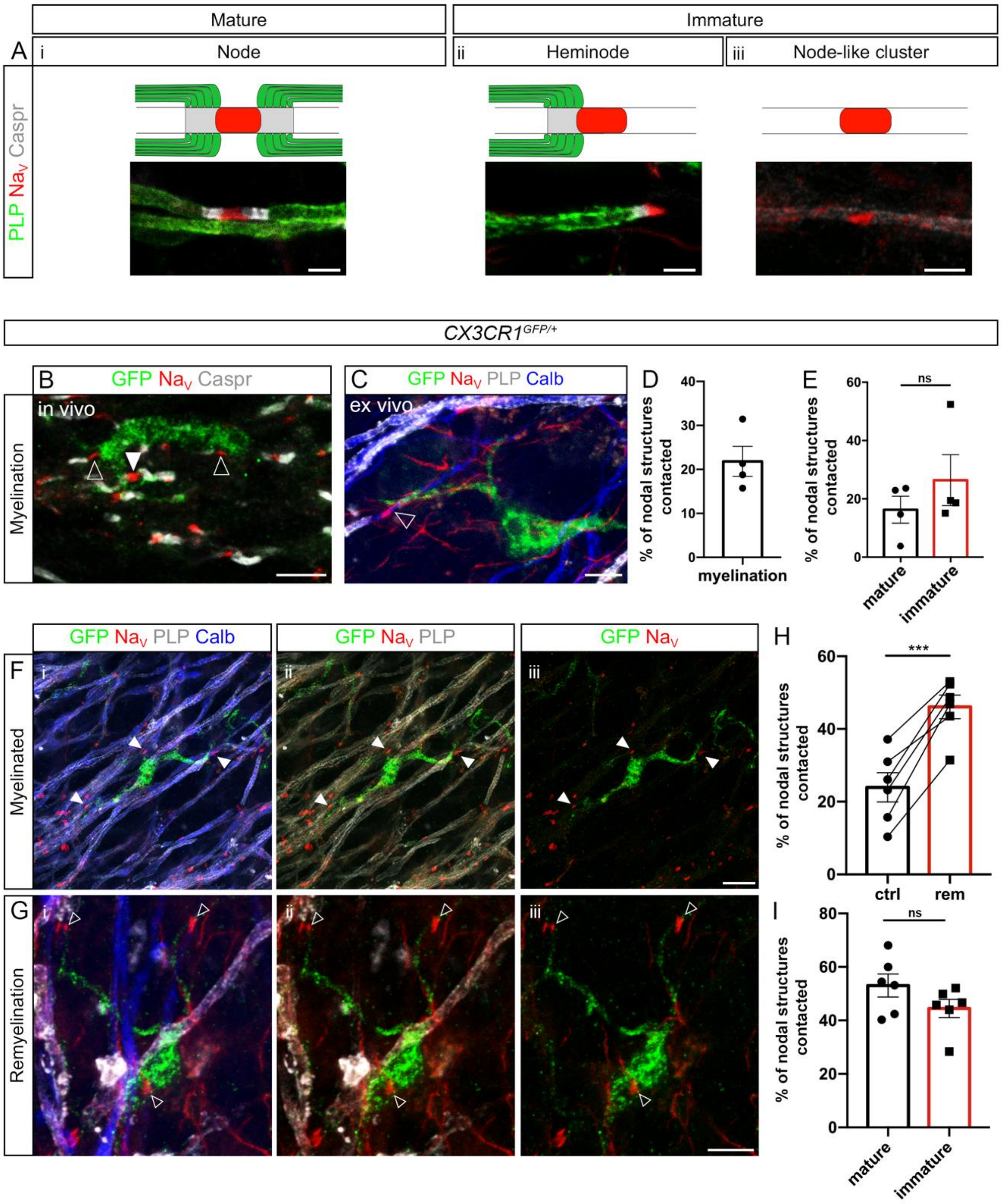

Figure S3. Microglial cells contact nodal structures in *ex vivo* organotypic cerebellar slice culture.

(A) Images and corresponding schematics illustrating a mature node (i) and immature nodal structures (heminode, ii and node-like-cluster, iii) in organotypic cerebellar slices (n=4 animals). (B, C) Microglia contact nodal structures during myelination in CX3CR1$^{GFP/+}$ mouse cerebellum *in vivo* (B, P12, n=3 animals) and *ex vivo* (C, 4 DIV, n=4 animals). Both mature nodes of Ranvier (filled arrowheads) and immature nodal structures (node-like clusters and

heminodes, empty arrowheads) are contacted. (D) Percentage of nodal structures contacted by microglia in myelinating slices *ex vivo* (n=4 animals). (E) Percentage of mature and immature nodal structures contacted in myelinating condition *ex vivo* (n=4 animals per condition). (F, G) Microglia also contact nodal structures in myelinated (F) and remyelinating slices (G) *ex vivo* (11 DIV). Arrowheads indicate the nodes of Ranvier contacted by microglia. (H) Percentage of nodal structures contacted by microglia in myelinated (ctrl) vs remyelinating (rem) slices. p= 0.0006 (I) Percentage of mature and immature nodal structures contacted in remyelinating condition (F-I: n=6 animals per condition). Scale bars: (A) 3 µm, (B, C) 5 µm, (F, G) 10 µm. (E) Two-sided Wilcoxon matched pairs test; (H, I) Two-sided Paired t-test. *P < 0.05, **P < 0.01, ***P < 0.001, ****P < 0.0001, ns: not significant; bars and error bars represent the mean ± s.e.m. For detailed statistics, see Supplementary Table.

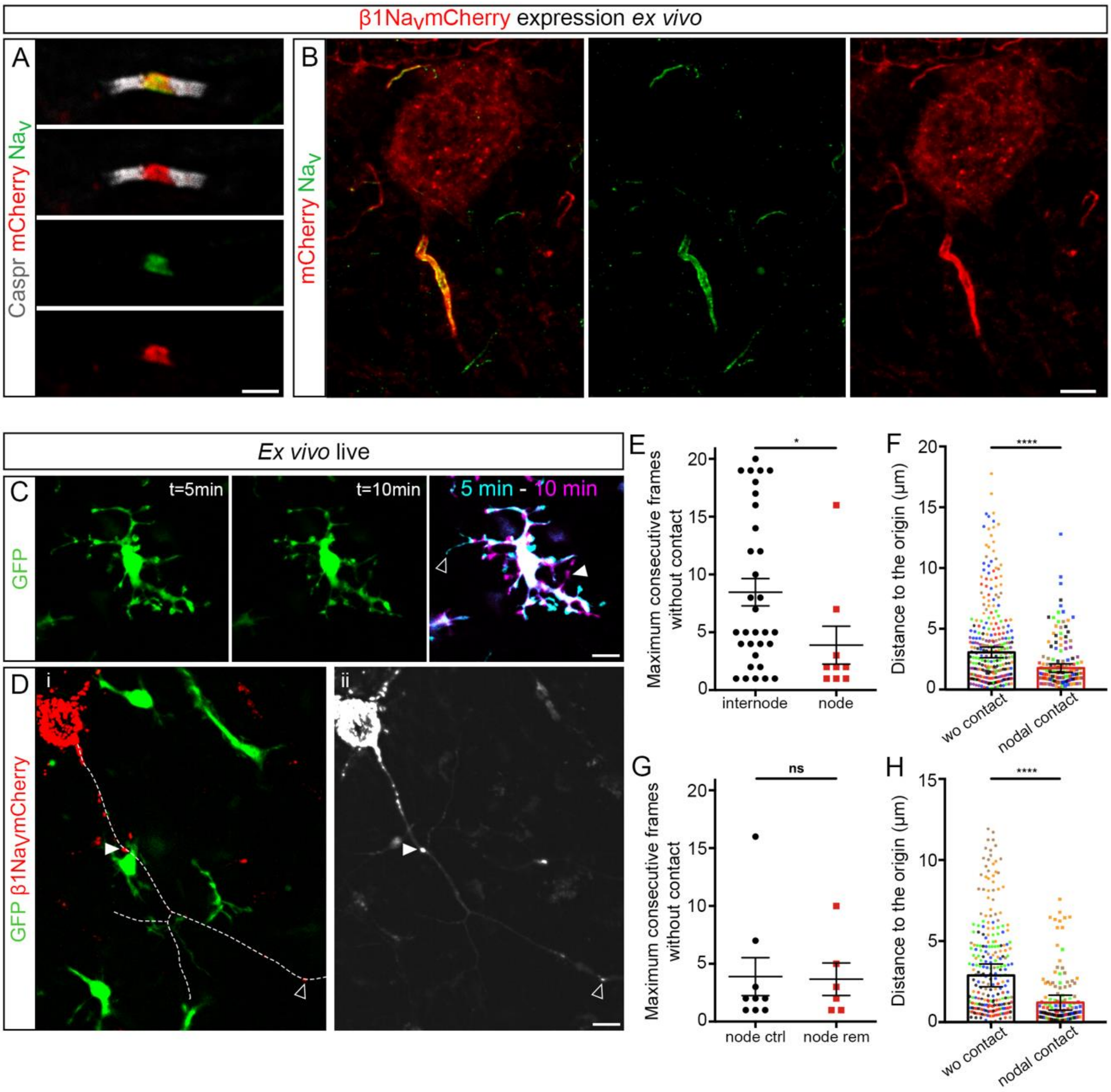

Figure S4. Live-imaging of microglia-node contact in organotypic cerebellar slice culture.

(A-B) β1Na$_V$mCherry axonal expression is restricted to the nodes of Ranvier (A) and AIS (B) in Purkinje neurons *ex vivo*, as shown by its colocalization with Na$_V$ (n=3 animals). (C) Live images of a microglial cell (GFP$^+$) at two different timepoints, and the corresponding overlay showing the dynamics of the microglial cell, with extending (magenta) and retracting (cyan) processes (arrowheads: most dynamic processes, n=59 microglial cells in n=6 animals). (D) Myelinated CX3CR1$^{GFP/+}$ cerebellar slice with a Purkinje cell expressing β1Na$_V$mCherry following lentiviral transduction. (i) Live image showing a node (mCherry$^+$, filled arrowhead) contacted by a microglial cell, and a non-contacted node (empty arrowhead). (ii) Summed projection of a movie showing the axon trajectory (n=24 animals). (E) Maximum duration

without contact between a microglial process and an internode or a node in myelinated slices (10 minutes acquisition, internode: n=32 contacts from 16 animals, node: n=9 contacts from 8 animals). (F) Distance between the process tip and its initial position for each frame, whether the process was initially contacting a node (nodal contact) or without contact (myelinated slices; wo contact: 280 measures from 14 trajectories, n=7 animals; nodal contact: 140 measures from 7 trajectories, n=7 color coded animals, p= 1.679e-08). (G) Maximum duration without contact between the tip of a microglial process and the node in myelinated (node ctrl) vs remyelinating (node rem) slices (10 minutes acquisition, ctrl: n=9 contacts from 8 animals, rem: n=6 contacts from 6 color coded animals). (H) Distance between the process tip and its initial position for each frame (remyelinating slices; wo contact: 240 measures from 12 trajectories, n=6 animals, nodal contact: 120 measures from 6 trajectories, n=6 animals, p= 2.2e-16). Scale bars: (A) 2 μm, (B) 5 μm, (C, D) 10 μm (E, G) Two-sided Mann-Whitney test; (F, H) Two-sided difference in means is indicated by Type II Wald chi-square test. *P < 0.05, **P < 0.01, ***P < 0.001, ****P < 0.0001, ns: not significant; bars and error bars represent the mean ± s.e.m. For detailed statistics, see Supplementary Table.

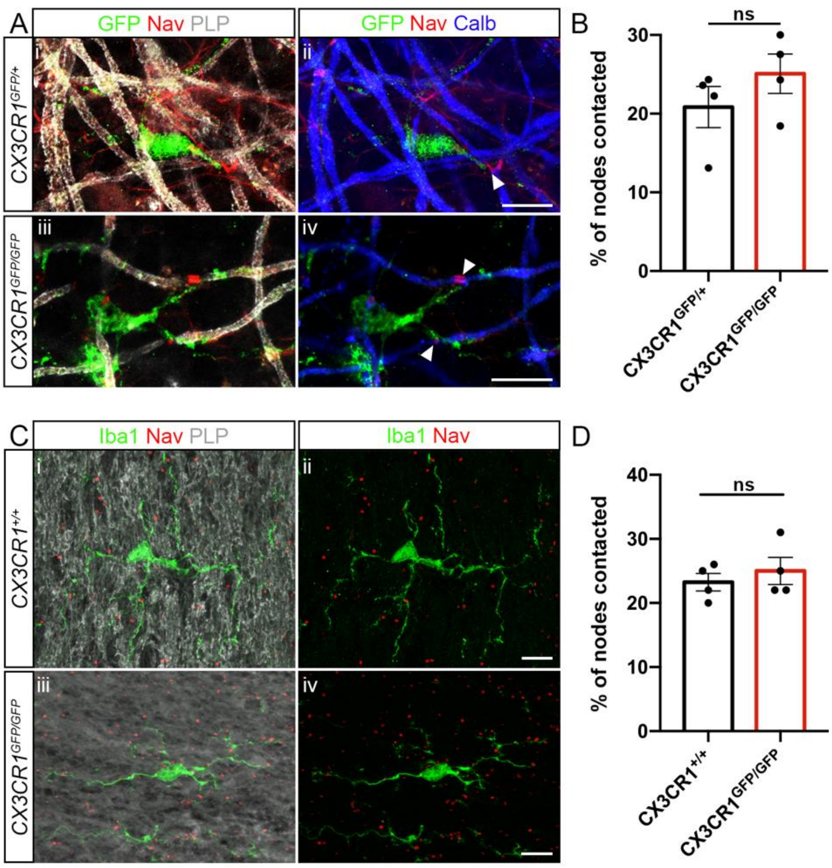

Figure S5. The microglial receptor CX3CR1 is not required for microglia-node interaction. (A) Microglia (GFP, in green) contact nodes of Ranvier (Na_V, in red) both in CX3CR1$^{GFP/+}$ and CX3CR1$^{GFP/GFP}$ myelinated cerebellar slices. (B) Percentage of nodes of Ranvier contacted by microglia in CX3CR1$^{GFP/+}$ vs CX3CR1$^{GFP/GFP}$ littermate slices (A-B, n=4 animals per condition). (C) Microglia (Iba1, in green) contact nodes (Na_V, in red) *in vivo* in CX3CR1$^{GFP/GFP}$ mouse spinal cord as in wild-type littermates. (D) Percentage of nodes of Ranvier contacted by microglia in CX3CR1$^{+/+}$ vs CX3CR1$^{GFP/GFP}$ littermate animals (C-D, n=4 animals per condition). Scale bars: (A, C) 10 μm. (B, D) Two-sided Mann-Whitney rank test. *$P < 0.05$, **$P < 0.01$, ***$P < 0.001$, ****$P < 0.0001$, ns: not significant; bars and error bars represent the mean ± s.e.m. For detailed statistics, see Supplementary Table.

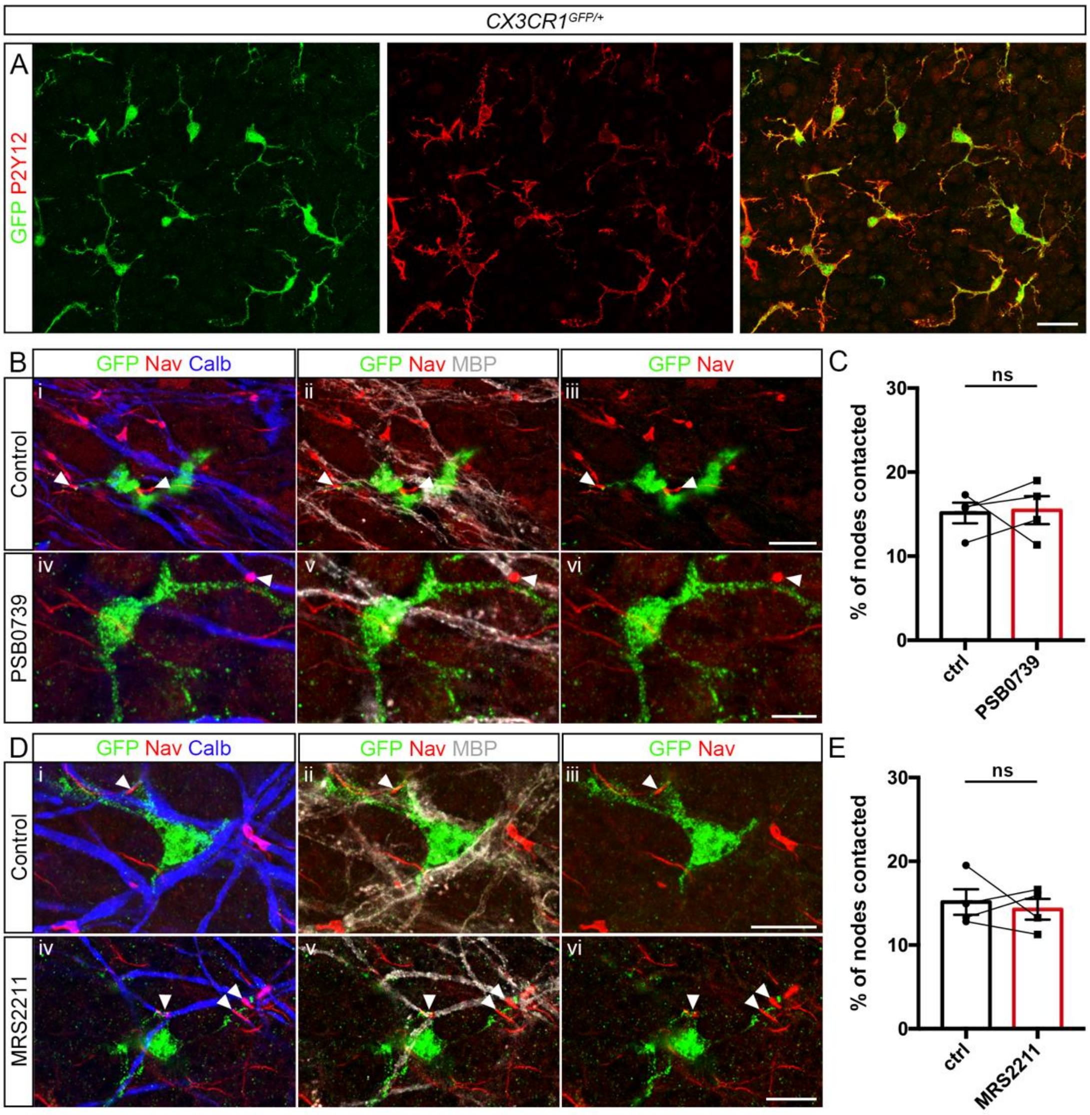

Figure S6. The microglial receptors P2Y12R and P2Y13R are not required for microglia-node interaction.

(A) Microglia express P2Y12R *ex vivo* in cerebellar organotypic cultures. (n=2 animals). (B, D) Illustration of microglia-node contacts in myelinated slices treated with PSB0739 (B, P2Y12R inhibitor, 1 µM, 3-hour treatment) or MRS2211 (D, P2Y12R/P2Y13R inhibitor 50 µM, 3-hours treatment). (C, E) Percentage of nodes contacted by microglial cells in *CX3CR1*$^{GFP/+}$ myelinated slices in control (ctrl) vs treated condition, with PSB0739 (C, 1 µM) or MRS2211 (E, 50 µM). (B-E): n=4 animals per condition. Arrowheads show the nodes of Ranvier contacted by microglial cells. Scale bars: (A) 20 µm, (Biii, D) 10 µm, (Bvi) 5 µm. (C, E) Two-sided Wilcoxon matched pairs test. *P < 0.05, **P < 0.01, ***P < 0.001, ****P < 0.0001, ns: not significant; bars and error bars represent the mean ± s.e.m. For detailed statistics, see Supplementary Table.

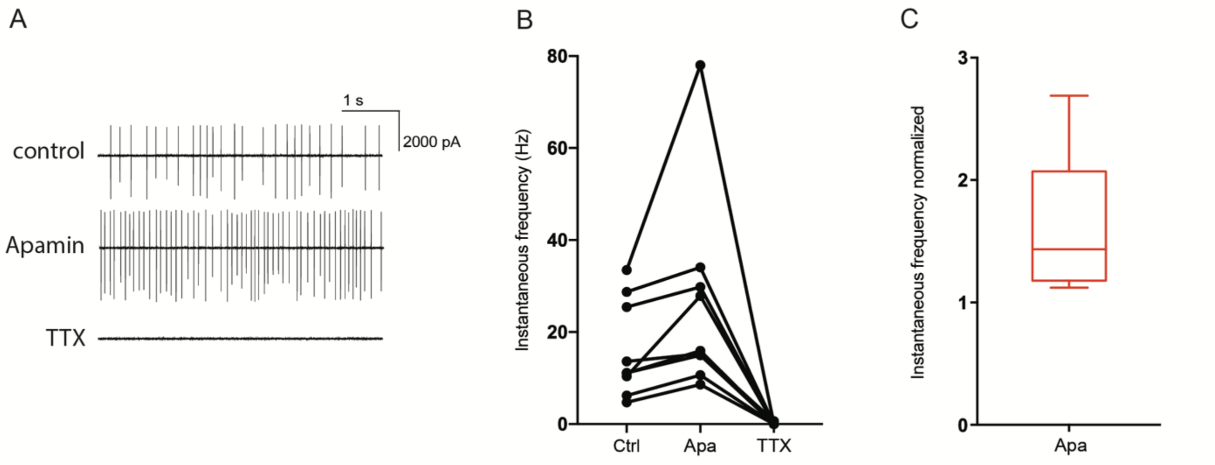

Figure S7. Purkinje cells present spontaneous activity in myelinated organotypic cerebellar slices.

(A) Representative example of loose-cell attached recordings on a Purkinje cell, in control condition followed by Apamin (Apa, 500 nM) and TTX (500 nM) consecutive treatments. (B) Instantaneous firing frequencies recorded in Purkinje cells of myelinated cerebellar organotypic slices (A-B, n=9 cells, n=7 animals, p=1.234e-4, Two-sided Friedman test). (C) Instantaneous firing frequencies recorded in Purkinje cells in myelinated cerebellar organotypic slices after Apamin treatment (500 nM), normalized by their matching control. The increased ratio of instantaneous frequencies ranges from 1.12 to 2.69, box-and-whiskers plot shows min, max, 25th and 75th percentiles and median (n=9 cells, n=7 animals). *P < 0.05, **P < 0.01, ***P < 0.001, ns: not significant; bars and error bars represent the mean ± s.e.m. For detailed statistics, see Supplementary Table.

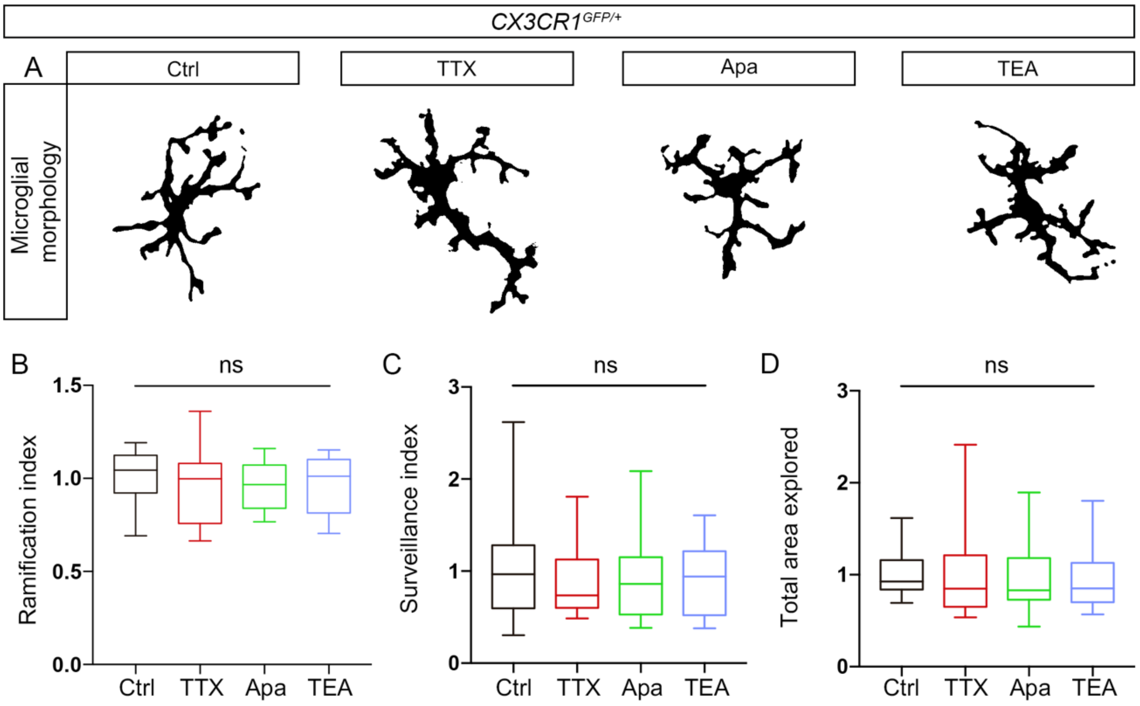

Figure S8. The dynamics and morphology of microglia are unchanged following TTX, Apamin and TEA treatments.

(A) Example of microglial morphology in control (ctrl) condition and following TTX, Apamin (Apa) or TEA treatments. (B-D) Microglia ramification (B) is unchanged after treatment, as well as their surveillance activity (C) and total area explored in 10 minute movies (D) (A-D, ctrl n=15 microglial cells, TTX: n=13 microglial cells, Apa n=15 microglial cells and TEA n=15 microglial cells, n=5 animals for each condition). Data are normalized by control values, box-and-whiskers plots show min, max, 25th and 75th percentiles and median. Two-sided Kruskal-Wallis test followed by Dunn's multiple comparison test, *P < 0.05, **P < 0.01, ***P < 0.001, ****P < 0.0001, ns: not significant; bars and error bars represent the mean ± s.e.m. For detailed statistics, see Supplementary Table.

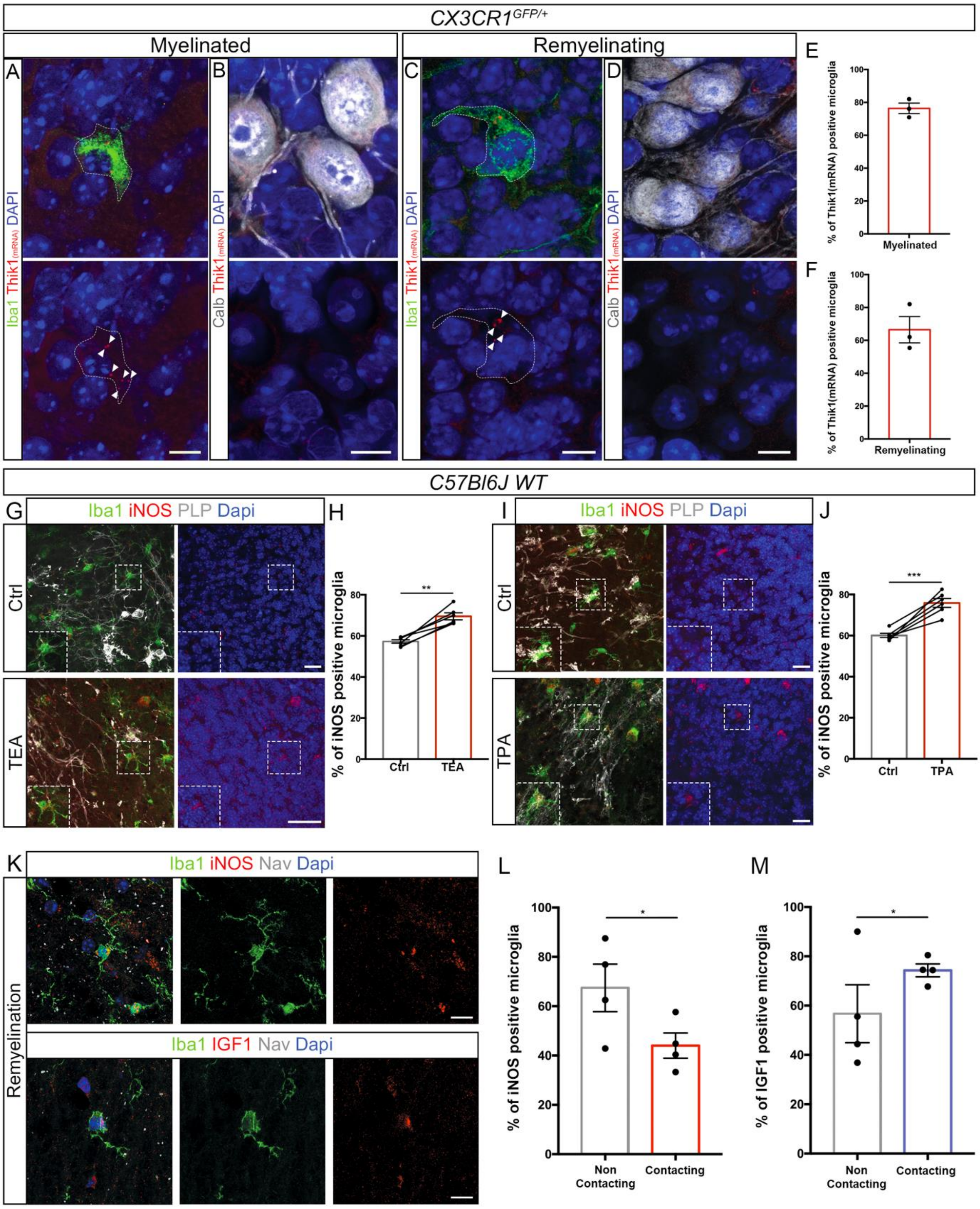

Figure S9. Microglia-node interaction influences microglial phenotype during remyelination. (A-F) THIK-1 mRNA (red) is expressed by microglia (Iba1, in green) but not Purkinje cells in myelinated as well as remyelinating organotypic cerebellar slices. Percentage of microglia with a clear expression of THIK-1 mRNA in myelinated and remyelinating slices. n=3 animals per condition. (G, I) In remyelinating C57bl6/J organotypic cerebellar slices, microglial expression

of iNOS is increased following neuronal potassium channel inhibition by TEA (G) or THIK-1 inhibition by TPA treatment (I). (H, J) Percentage of iNOS+ microglial cells at remyelination onset, with no treatment (ctrl) or with TEA (H, 2 hours, 30 mM) or TPA treatment (J, 2 hours, 50 μM). The mean values per animal are individually plotted and paired with the corresponding control. (G-J, n=6 animals per condition). (K) Both iNOS+ and IGF1+ microglial cells contact nodes of Ranvier in remyelinating mouse dorsal spinal cord *in vivo*. (L, M) Percentage of iNOS (L) and IGF1 (M) positive microglial cells amongst "contacting" or "non-contacting" microglial cells. The mean values per animal are shown as dots (K-M, n=6 animals per condition). Scale bars: (A-D) 10 μm, (G, I) 50 μm, (K) 10 μm; (H, J) Two-sided Paired t-test. (L, M) Two-sided Cochran-Mantel-Haenszel chi-squared test. *P < 0.05, **P < 0.01, ***P < 0.001, ****P < 0.0001, ns: not significant; bars and error bars represent the mean ± s.e.m. For detailed statistics, see Supplementary Table.
