# Supplementary material for: Microglia-neuron interaction at nodes of Ranvier depends on neuronal activity through potassium release and contributes to remyelination
Source: Nat Commun. 2021 Sep 1;12:5219. doi: 10.1038/s41467-021-25486-7 (PMC8410814; doi:10.1038/s41467-021-25486-7)
Supplement: Supplementary file 3 — Description of Additional Supplementary Files [file 41467_2021_25486_MOESM3_ESM.docx]

**Description of Additional Supplementary Files**

**Title: Supplementary Movie 1 (from Fig3). Stable interaction between a node and a microglial cell along time.**

Description: Representative example of a three-hour movie from a CX3CR1-GFP/Thy1-Nfasc186-mCherry mouse showing a durable interaction between a microglial cell (green) and a node of Ranvier (red). *In vivo* acquisitions every 30 minutes. Sham animal (NaCl injection) DPI 11. Filled arrowheads indicate contact, empty arrowheads an absence of contact. Scale bar: 10µm.

**Title: Supplementary Movie 2 (from FigS2).** **Stable interaction between a node and a microglial cell in control condition.**

Description: Representative example of a one-hour movie from a CX3CR1-GFP/Thy1-Nfasc186-mCherry mouse allowing to observe interactions between microglia in green and nodes of Ranvier in red. *In vivo* acquisitions every 10 minutes. Sham animal (NaCl injection) DPI 7. Filled arrowheads indicate contact, empty arrowheads an absence of contact. Scale bar: 10µm.

**Title: Supplementary Movie 3 (from FigS2). Instable interaction between a node and a microglial cell in the perilesional tissue in demyelinating context.**

Description: Representative example of a one-hour movie from a CX3CR1-GFP/Thy1-Nfasc186-mCherry mouse allowing to observe the intermittent interaction between microglia in green and nodes of Ranvier in red. *In vivo* acquisitions every 10 minutes. LPC injection DPI 7 (perilesional area, peak of demyelination). Filled arrowheads indicate contact, empty arrowheads an absence of contact. Scale bar: 10µm.

**Title: Supplementary Movie 4 (from FigS2).** **Stable interaction between a node and a microglial cell during remyelination.**

Description: Representative example of a one-hour movie from a CX3CR1-GFP/Thy1-Nfasc186-mCherry mouse allowing to observe stable interactions between microglia in green and nodes of Ranvier in red. *In vivo* acquisitions every 10 minutes. LPC injection DPI 11 (remyelination). Filled arrowheads indicate contact, empty arrowheads an absence of contact. Scale bar: 10µm.

**Title: Supplementary Movie 5 (from Fig4). A microglial process tip contacting an internode in a myelinated slice.**

Description: Representative example showing a microglial cell (green) initially contacting an internode (red) in a myelinated slice. Filled arrowheads indicate a contact, and empty arrowheads an absence of contact. The dashed line represents the axon. Scale bar: 5µm.

**Title: Supplementary Movie 6 (from Fig4). A microglia process tip contacting a node in a myelinated slice.**

Description: Representative example showing a microglial cell (green) initially contacting a node (red) in a myelinated slice. Filled arrowheads indicate a contact, empty arrowheads an absence of contact. The dashed line represents the axon. Scale bar: 5µm.

**Title: Supplementary Movie 7 (from Fig4). A microglia process tip contacting a node in a remyelinating slice.**

Description: Representative example showing a microglial cell (green) initially contacting a node (red) in a remyelinating slice. Filled arrowheads indicate a contact, empty arrowheads an absence of contact. The dashed line represents the axon. Scale bar: 5µm.

**Title: Supplementary Movie 8 (from Fig6). Microglia process tip contacting a node in a myelinated slice treated with TEA.**

Description: Representative example showing a microglial cell (green) initially contacting a node (red) in a myelinated slice treated with TEA. Filled arrowheads indicate a contact, empty arrowheads an absence of contact. The dashed line represents the axon. Scale bar: 5µm.
