# Supplementary material for: Microglia-neuron interaction at nodes of Ranvier depends on neuronal activity through potassium release and contributes to remyelination
Source: Nat Commun. 2021 Sep 1;12:5219. doi: 10.1038/s41467-021-25486-7 (PMC8410814; doi:10.1038/s41467-021-25486-7)
Supplement: Supplementary file 12 — Reporting Summary [file 41467_2021_25486_MOESM12_ESM.pdf]

nature research

Corresponding author(s): Desmazieres Anne
Lubetzki Catherine

Last updated by author(s): 20/07/2021

Reporting Summary

Nature Research wishes to improve the reproducibility of the work that we publish. This form provides structure for consistency and transparency in reporting. For further information on Nature Research policies, see our Editorial Policies and the Editorial Policy Checklist.

Statistics

For all statistical analyses, confirm that the following items are present in the figure legend, table legend, main text, or Methods section.

n/a	Confirmed	
☐	☒	The exact sample size (*n*) for each experimental group/condition, given as a discrete number and unit of measurement
☐	☒	A statement on whether measurements were taken from distinct samples or whether the same sample was measured repeatedly
☐	☒	The statistical test(s) used AND whether they are one- or two-sided<br>*Only common tests should be described solely by name; describe more complex techniques in the Methods section.*
☐	☒	A description of all covariates tested
☐	☒	A description of any assumptions or corrections, such as tests of normality and adjustment for multiple comparisons
☐	☒	A full description of the statistical parameters including central tendency (e.g. means) or other basic estimates (e.g. regression coefficient) AND variation (e.g. standard deviation) or associated estimates of uncertainty (e.g. confidence intervals)
☐	☒	For null hypothesis testing, the test statistic (e.g. *F*, *t*, *r*) with confidence intervals, effect sizes, degrees of freedom and *P* value noted<br>*Give P values as exact values whenever suitable.*
☒	☐	For Bayesian analysis, information on the choice of priors and Markov chain Monte Carlo settings
☒	☐	For hierarchical and complex designs, identification of the appropriate level for tests and full reporting of outcomes
☒	☐	Estimates of effect sizes (e.g. Cohen's *d*, Pearson's *r*), indicating how they were calculated

Our web collection on statistics for biologists contains articles on many of the points above.

Software and code

Policy information about availability of computer code

Data collection	LAS X Software (Leica, v 3.5.6), Metamorph (version 7.8.13.0)
Data analysis	Images processing and analysis were performed using: ImageJ (NIH, Bethesda, Maryland, version 1.52p); Zen (Zeiss, 2010, version 14.0.23.201); Imaris software (GraphPad, Bitplane, version 9.2); Huygens software (version 17.10). Statistical analysis were performed using R (version 3.5.2) and Prism (version 7.0). Figures and schematics were made using Adobe Photoshop (version CC 2019) and Adobe Illustrator (version CC 2019)

For manuscripts utilizing custom algorithms or software that are central to the research but not yet described in published literature, software must be made available to editors and reviewers. We strongly encourage code deposition in a community repository (e.g. GitHub). See the Nature Research guidelines for submitting code & software for further information.

Data

Policy information about availability of data

All manuscripts must include a data availability statement. This statement should provide the following information, where applicable:

- Accession codes, unique identifiers, or web links for publicly available datasets
- A list of figures that have associated raw data
- A description of any restrictions on data availability

All data are available in the manuscript. Raw source files and scripts for remyelination analysis (ImajeJ), data representation (R software and Prism) and statistical analysis (R software and Prism) are available upon request. Detailled plasmid description is available upon request.

Field-specific reporting

Please select the one below that is the best fit for your research. If you are not sure, read the appropriate sections before making your selection.

- [x] Life sciences
- [ ] Behavioural & social sciences
- [ ] Ecological, evolutionary & environmental sciences

For a reference copy of the document with all sections, see nature.com/documents/nr-reporting-summary-flat.pdf

Life sciences study design

All studies must disclose on these points even when the disclosure is negative.

Sample size	Sample size was initially defined in accordance with previously published experiments using similar experimental design (14, 16, 30, 60, 64).
Data exclusions	One sample was formally excluded from analysis by Grubb's outlier test.
Replication	Experiments were replicated multiple times, with the number of replication clearly indicated in the text or figures for quantified parameters. When descriptive data are presented without quantification, the experiment were replicated at least three times (biological replicates) and all attempts at replication were sucessful.
Randomization	Batches of animals from different litters/parents were used to replicate experiments. The animals were then randomly allocated to each experimental groups.
Blinding	For the initial experiments addressing the modulation of microglia-node interactions by neuronal activity, TEA and TPA treatments, we performed a blinded analysis. In control versus demyelinated tissues, regarding microglial phenotypes and myelin extent, the quantified parameters were strictly defined prior to quantification to avoid any bias, as differences were too visible to perform a blinded experiment. All automated analysis were not blinded.

Reporting for specific materials, systems and methods

We require information from authors about some types of materials, experimental systems and methods used in many studies. Here, indicate whether each material, system or method listed is relevant to your study. If you are not sure if a list item applies to your research, read the appropriate section before selecting a response.

Materials & experimental systems

n/a	Involved in the study	
[ ]	[x]	Antibodies
[x]	[ ]	Eukaryotic cell lines
[x]	[ ]	Palaeontology and archaeology
[ ]	[x]	Animals and other organisms
[ ]	[x]	Human research participants
[x]	[ ]	Clinical data
[x]	[ ]	Dual use research of concern

Methods

n/a	Involved in the study	
[x]	[ ]	ChIP-seq
[x]	[ ]	Flow cytometry
[x]	[ ]	MRI-based neuroimaging

Antibodies

Antibodies used	Primary Antibodies: mouse IgG2a anti-AnkyrinG (clone N106/36; 1:100; cat# 75-146), mouse IgG2b anti-AnkyrinG (clone N106/65; 1:75; cat# 75-147) and mouse IgG1 anti-Caspr (clone K65/35; 1:100; cat# 10671175), all from Neuromab. Mouse IgG1 anti-Pan Nav (clone K58/35; 1:150; Sigma; cat# S8809); mouse IgG1 anti-Calbindin (1:500; Sigma; cat# C9848), rabbit anti-Calbindin (1:500; Swant; cat# CB38), rabbit anti-Caspr (1:300; Abcam; cat# ab34151), rat anti-PLP (1:10; generated by and to be requested to Dr. K. Ikenaka, Okasaki, Japan), mouse IgG2b anti-MBP (1:200; SMI99; Sigma; cat# NE1019), rabbit IgG anti-Iba1 (1:500; Wako; cat# 019-19741), chicken anti-GFAP (1:500; Aves Labs; cat# AB_2313547), rat anti-PDGFRα (1:100; BD Biosciences; cat# 558774), rabbit IgG anti-TMEM119 (1:100; Sigma cat# HPA051870), rabbit IgG anti-P2Y12r (1:300; Alomone; cat# APR-012), rabbit anti-P2Y12R (1:300; Anaspec; cat# AS-55043A), chicken anti-mCherry (1:1000; Abcam; cat# ab205402), chicken anti-GFP (1:250; Millipore; cat# 06-896), mouse IgG2a anti-iNOS (1:100; BD Biosciences; cat# 610328), goat anti-IGF1 (1:50; R&D System; cat# AF791).<br>Fluorescent secondary antibodies : goat or donkey anti-chicken, goat, mouse IgG2a, IgG2b, IgG1, rabbit and rat coupled to Alexa Fluor 488, 594, 647 or 405 from Invitrogen (1:500), or goat anti-mouse IgG1 DyLight (1:600; Jackson Immuno Research; cat# 115-477-187).<br>Biotinylated secondary antibody: biotinylated goat anti-rabbit IgG (1:200; Vactor Labs; cat# BA-1000-1.5)
Validation	Each antibody was validated for the species (mouse or human) and application (immunohistochemistry) by the correspondent manufacturer or/and within the lab. Their use was described in the methods section of the manuscript.

Animals and other organisms

Policy information about studies involving animals; ARRIVE guidelines recommended for reporting animal research

Laboratory animals	C57bl6/J (Post natal day 9 and 10, 2-4 months adults), CX3CR1-GFP (Post natal day 9 and 10) and Cx3CR1-GFP/Thy1Nfasc186mcherry were used at adult ages (2-4 months) for in vivo studies; C57bl6/J and CX3CR1-GFP were used at P9-P10 for ex vivo studies. The animals were housed in an enriched environment, at 22+/-2°C, in ventilated cages (6 adult animals maximum per cage) under a light/ dark cycle of 12 hours.
Wild animals	No wild animals were used in the study.
Field-collected samples	No field collected samples were used in the study.
Ethics oversight	The care and use of mice were conformed to institutional policies and guidelines (UPMC, INSERM, French and European Community Council Directive 86/609/EEC)

Note that full information on the approval of the study protocol must also be provided in the manuscript.

Human research participants

Policy information about studies involving human research participants

Population characteristics	Post-mortem tissues were from 3 female and 2 male control patients, mean age 71,6 (60-85)
Recruitment	UK MS Society Tissue Bank at Imperial College, London
Ethics oversight	Under ethical approval by the National Research Ethics Committee 08/MRE09/31

Note that full information on the approval of the study protocol must also be provided in the manuscript.
